# Machine Learning Model-Based Prediction of In-Hospital Acute Kidney Injury Risk in Acute Aortic Dissection Patients

**DOI:** 10.31083/RCM25768

**Published:** 2025-02-21

**Authors:** Zhili Wei, Shidong Liu, Yang Chen, Hongxu Liu, Guangzu Liu, Yuan Hu, Bing Song

**Affiliations:** ^1^The First Clinical Medical College of Lanzhou University, 730000 Lanzhou, Gansu, China; ^2^Department of Cardiovascular Surgery, First Hospital of Lanzhou University, 730000 Lanzhou, Gansu, China

**Keywords:** acute aortic dissection, acute kidney injury, machine learning, prediction model

## Abstract

**Background:**

This study aimed to identify the risk factors for in-hospital acute kidney injury (AKI) in patients with acute aortic dissection (AAD) and to establish a machine learning model for predicting in-hospital AKI.

**Methods:**

We extracted data on patients with AAD from the Medical Information Mart for Intensive Care (MIMIC)-IV database and developed seven machine learning models: support vector machine (SVM), gradient boosting machine (GBM), neural network (NNET), eXtreme gradient boosting (XGBoost), K-nearest neighbors (KNN), light gradient boosting machine (LightGBM), and categorical boosting (CatBoost). Model performance was assessed using the area under the receiver operating characteristic curve (AUC), and the optimal model was interpreted using Shapley Additive explanations (SHAP) visualization analysis.

**Results:**

A total of 325 patients with AAD were identified from the MIMIC-IV database, of which 84 patients (25.85%) developed in-hospital AKI. This study collected 42 features, with nine selected for model building. A total of 70% of the patients were randomly allocated to the training set, while the remaining 30% were allocated to the test set. Machine learning models were built on the training set and validated using the test set. In addition, we collected AAD patient data from the MIMIC-III database for external validation. Among the seven machine learning models, the CatBoost model performed the best, with an AUC of 0.876 in the training set and 0.723 in the test set. CatBoost also performed strongly during the validation, achieving an AUC of 0.712. SHAP visualization analysis identified the most important risk factors for in-hospital AKI in AAD patients as maximum blood urea nitrogen (BUN), body mass index (BMI), urine output, maximum glucose (GLU), minimum BUN, minimum creatinine, maximum creatinine, weight and acute physiology score III (APSIII).

**Conclusions:**

The CatBoost model, constructed using risk factors including maximum and minimum BUN levels, BMI, urine output, and maximum GLU, effectively predicts the risk of in-hospital AKI in AAD patients and shows compelling results in further validations.

## 1. Introduction

Acute aortic dissection (AAD) is characterized by a tear in the aortic intima, with symptoms manifesting within two weeks. Blood flows through this tear into the middle layer of the aorta, forming a true lumen and a false lumen, progressively separating the inner and middle layers of the aorta [1]. Currently, AAD is primarily classified into two types: Stanford type A, which involves the ascending aorta, and Stanford type B, which does not [2]. Clinically, AAD typically presents with acute, severe chest and back pain and is characterized by rapid onset, swift progression, diverse initial symptoms, and high mortality risk [3]. The incidence rate of AAD is about 0.005%, but the mortality rate within 24 hours can reach 33%. Without timely intervention, the mortality rate increases cumulatively by 0.5% each hour, reaching 50% within 48 hours and 74% within one week [4, 5]. The primary treatments for AAD include surgical repair and endovascular treatment, which have been demonstrated to achieve survival rates of up to 90% when timely administered [6]. Acute kidney injury (AKI) is a common complication among AAD patients, occurring either in-hospital or post-surgery, with an incidence rate of 7%–20% [7, 8]. The occurrence of AKI in AAD patients often exacerbates the condition, leading to further complications, prolonged hospital stays, and increased mortality rates [9]. Consequently, it is crucial to establish a robust predictive model for effectively forecasting AKI in hospitalized AAD patients.

Recently, the application of artificial intelligence in the medical field has become increasingly widespread. Machine learning (ML), an important branch of artificial intelligence [10, 11], delves deeper into the intrinsic patterns of data when faced with highly complex, high-dimensional clinical data compared to traditional prediction models. Prediction models developed using machine learning and now widely utilized in clinical predictions exhibit greater stability, higher accuracy, and stronger generalization capabilities [12, 13]. The main ML types include supervised learning, unsupervised learning, and others [14]. The Medical Information Mart for Intensive Care (MIMIC)-IV database is a commonly used large single-center database that contains the clinical data of 382,278 patients at Beth Israel Deaconess Medical Center from 2008 to 2019. The data include demographic characteristics, radiological examination results, laboratory test results, patient vital signs, medication treatment data, various scoring data, in-hospital complications, and clinical outcomes [15, 16]. The MIMIC database is widely used due to its high-quality and comprehensive data records. Previous literature has established a predictive model for in-hospital mortality of AAD patients [17, 18] and reports of predictive models for AKI following acute myocardial infarction [19]; however, there is currently no research on machine learning models related to in-hospital AKI complications in AAD patients. Therefore, this study extracted clinical data of AAD patients from the MIMIC-IV database to establish seven types of machine learning models: support vector machine (SVM), gradient boosting machine (GBM), neural network (NNET), eXtreme gradient boosting (XGBoost), K-nearest neighbors (KNN), light gradient boosting machine (LightGBM), and categorical boosting (CatBoost). Simultaneously, the AAD patient data in the MIMIC-III database were used to externally validate the established optimal model. These machine learning models are employed to screen for risk factors and predict in-hospital AKI complications in AAD patients, aiding clinical decision-making.

## 2. Materials and Methods

### 2.1 Clinical Data Source

The patients studied primarily originated from Version 2.2 of the MIMIC-IV and Version 1.4 of the MIMIC-III databases, which mainly include records from the Beth Israel Deaconess Medical Center from 2001 to 2019. The study team obtained specific approval and permission for the data retrieval process. Data extraction was primarily conducted using structured query language (SQL) and Navicat Premium version16.0 (PremiumSoft Cyber Tech, Hong Kong, China). Since all patient data in the database were anonymized, no additional ethical approval was required for this study.

### 2.2 Data Collection

Inclusion criteria: patients diagnosed with AAD according to the International Classification of Diseases (ICD) 9th and 10th editions, with ICD-9 diagnostic codes: 441.01, 441.02, 441.03; ICD-10 diagnostic codes: I71.01, I71.02, I71.03. Exclusion criteria: (1) patients with an intensive care unit (ICU) stay of less than 24 hours; (2) patients with repeated hospital admissions or ICU readmissions; (3) patients with a history of renal-related diseases; (4) patients aged under 18 years; (5) patients with no surgical treatment or only minimally invasive surgery. Finally, following the strict inclusion and exclusion criteria, we collected 325 patients from the MIMIC-IV database and 179 patients from the MIMIC-III database. We collected data on AAD patients within 24 hours of their admission to the ICU. The data collected in this study included: (1) demographic characteristics: gender, age, height, weight, body mass index (BMI); (2) laboratory test data: hemoglobin (HB), platelet (PLT), white blood cells (WBCs), anion gap (AG), bicarbonate (BC), blood urea nitrogen (BUN), creatinine, blood glucose (GLU), calcium (Ca) ions, sodium (Na) ions, potassium (K) ions, international normalized ratio (INR); (3) various scores: Acute Physiology Score III (APSIII), Sequential Organ Failure Assessment score (SOFA score), Charlson comorbidity index, Glasgow coma scale score (GCS score); (4) vital signs: urine output, systolic blood pressure, diastolic blood pressure; (5) other data: overall length of stay (LOS), LOS in the ICU, and number of deaths.

### 2.3 Model Establishment and Evaluation

Both univariate (single-factor) and multivariate (multi-factor) logistic regression analyses were performed on the training dataset to identify and utilize risk factors for in-hospital AKI in AAD patients for model construction. This study established seven types of machine learning models: SVM model, GBM model, NNET model, XGBoost model, KNN model, LightGBM model, and CatBoost model. The models were developed using the training set, and their performance was enhanced through 10-fold cross-validation. Feature importance ranking and other model evaluation metrics were employed for accuracy, sensitivity, specificity, precision, and the F1 score. We used receiver operating characteristic (ROC) curves, decision curve analysis (DCA) curves and precision-recall (PR) curves to evaluate the performance of the model. In addition, we used data obtained from the MIMIC-III database of 179 patients to validate the model externally. Finally, Shapley Additive explanations (SHAP) visualizations were utilized to interpret the optimal model, providing insights into the decision-making processes of the model.

### 2.4 Outcome Measures 

The outcome measure for this study is the new onset of AKI during hospitalization, according to the current international diagnostic criteria for AKI [20]: (1) a rise in serum creatinine by ≥0.3 mg/dL or ≥26.5 µmol/L within 48 hours; (2) a rise in baseline serum creatinine by at least 50% within 7 days; (3) urinary output <0.5 mL/kg/h within 6 hours.

### 2.5 Statistical Analysis

First, the data were preprocessed by removing features with more than 20% missing values, and the remaining missing values were added to the dataset using the predictive mean matching method (PMM) for multiple imputations. PMM primarily uses the other feature values of a sample to predict the missing values. After imputation, numerous datasets are generated, and researchers could choose one for further data analysis, removing duplicates and samples containing outliers. Following the data preprocessing, the final cohort of 325 patients was randomly divided into a training set (228 patients) and a test set (97 patients) in a 7:3 ratio. Since there are only 59 AKI patients in the training set (25.88%), the large disparity between AKI and non-AKI patients had the potential to reduce the performance of the model. We employed the synthetic minority oversampling technique (SMOTE) oversampling technique to create a more balanced representation between AKI and non-AKI patient cases. The mean ± standard deviation represents continuous variables, and categorical variables are represented by frequency (rate). Continuous variables were tested for comparisons using the *t*-test if normally distributed or with the Mann–Whitney U test if not. Categorical variables were tested using the chi-square test. This study utilizes R 4.3.2 (R Foundation for Statistical Computing, Vienna, Austria) and Python 3.10 (Python Software Foundation, Austin, TX, USA) for data analysis and chart creation, with statistical significance at *p* < 0.05.

## 3. Results

### 3.1 Baseline Characteristics

**Fig. 1.**
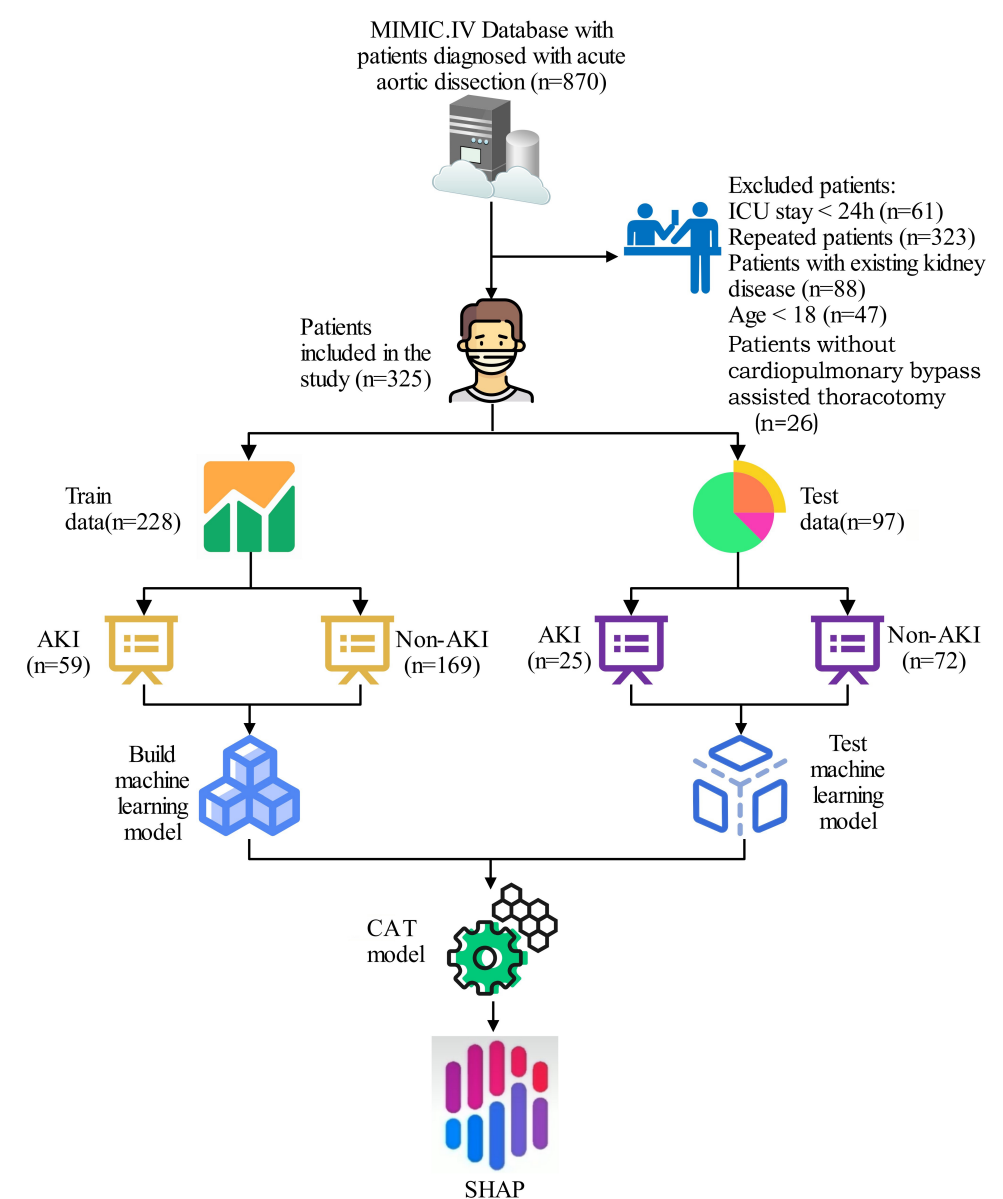
**Patient screening process diagram**. AKI, acute kidney injury; ICU, intensive care unit; SHAP, Shapley Additive explanations; CAT, categorical boosting; MIMIC, Medical Information Mart for Intensive Care.

This study included 325 AAD patients from the MIMIC-IV database, 84 of whom were AKI patients. Significant differences were observed between the AKI and non-AKI groups in several parameters: weight, BMI, APSIII, minimum BUN, maximum BUN, maximum creatinine, minimum creatinine, maximum glucose, and urine output (*p* < 0.05). The two groups had no statistical differences in other characteristics (*p* > 0.05). Fig. 1 presents the patient screening flowchart, Table 1 presents the baseline characteristics of the patients in the training set in detail, and Table 2 presents the baseline characteristics of the patients in the validation set.

**Table 1.** **Univariate analysis results of the training set**.

Variables		Non-AKI (n = 169)	AKI (n = 59)	*p*-value
Gender (male) (n (%))		129 (76.3)	49 (83.1)	0.373
Procedure (yes) (n (%))				
	Extracorporeal circulation auxiliary to open heart surgery	169 (100)	59 (100)	-
	Resection of vessel with replacement, thoracic vessels	142 (80.4)	53 (89.8)	0.381
	Open heart valvuloplasty of aortic valve without replacement	34 (20.1)	9 (15.3)	0.529
	Open and other replacement of aortic valve with tissue graft	31 (18.3)	12 (20.3)	0.885
	Endovascular implantation of graft in thoracic aorta	28 (16.6)	8 (13.6)	0.735
Age (years)		68.27 ± 15.50	68.52 ± 12.58	0.910
Height (cm)		169.22 ± 11.70	169.88 ± 11.16	0.708
Weight (kg)		80.56 ± 20.78	91.43 ± 24.60	0.001
BMI		23.71 ± 5.52	26.82 ± 6.56	<0.001
APSIII		40.28 ± 17.21	51.08 ± 18.84	<0.001
GCS score		13.96 ± 2.66	14.22 ± 2.27	0.501
Charlson comorbidity index		4.60 ± 2.41	5.05 ± 2.40	0.221
SOFA score		4.78 ± 3.65	5.80 ± 3.63	0.065
HB min (g/dL)		9.68 ± 2.34	9.28 ± 2.30	0.248
HB max (g/dL)		11.84 ± 1.96	12.10 ± 2.22	0.393
PLT min (×10^9^/L)		172.37 ± 100.31	153.76 ± 76.98	0.196
PLT max (×10^9^/L)		218.14 ± 103.39	213.00 ± 80.40	0.729
WBC min (×10^9^/L)		9.19 ± 8.85	9.12 ± 3.47	0.948
WBC max (×10^9^/L)		13.93 ± 19.70	13.86 ± 5.40	0.981
AG min (mmol/L)		11.85 ± 2.76	12.32 ± 2.90	0.268
AG max (mmol/L)		15.00 ± 3.54	16.05 ± 3.66	0.053
BC min (mmol/L)		22.44 ± 3.69	22.05 ± 3.42	0.473
BC max (mmol/L)		25.09 ± 3.34	25.49 ± 3.20	0.428
BUN min (mmol/L)		18.78 ± 13.03	24.81 ± 17.72	0.006
BUN max (mmol/L)		21.67 ± 14.57	31.37 ± 21.68	<0.001
Ca min (mmol/L)		8.30 ± 0.83	8.33 ± 0.93	0.676
Ca max (mmol/L)		8.76 ± 0.83	8.78 ± 0.80	0.855
Creatinine min (mg/dL)		1.03 ± 0.70	1.52 ± 1.50	0.001
Creatinine max (mg/dL)		1.20 ± 0.75	2.04 ± 2.07	<0.001
GLU min (mmol/L)		113.50 ± 31.62	120.88 ± 36.77	0.141
GLU max (mmol/L)		143.50 ± 53.39	166.41 ± 70.45	0.010
Na min (mmol/L)		137.94 ± 4.19	137.34 ± 3.33	0.320
Na max (mmol/L)		140.24 ± 3.74	140.93 ± 2.99	0.202
K min (mmol/L)		3.86 ± 0.49	3.97 ± 0.59	0.159
K max (mmol/L)		4.51 ± 0.76	4.74 ± 0.92	0.065
INR min		1.26 ± 0.36	1.24 ± 0.35	0.807
INR max		1.61 ± 1.16	1.61 ± 0.56	0.998
Urine output (mL)		1563.87 ± 893.49	1244.14 ± 843.13	0.017
SBP min (mmHg)		90.41 ± 15.35	87.47 ± 14.73	0.202
SBP max (mmHg)		149.23 ± 20.53	144.73 ± 17.46	0.134
DBP min (mmHg)		44.54 ± 9.83	44.17 ± 9.09	0.800
DBP max (mmHg)		82.70 ± 17.95	82.46 ± 15.32	0.927
Length of stay (days)		10.68 ± 9.49	13.49 ± 11.06	0.062
LOS in ICU (days)		5.99 ± 7.37	8.04 ± 9.86	0.096

AKI, acute kidney injury; BMI, body mass index; APSIII score, Acute Physiology Score III; GCS score, Glasgow coma scale score; SOFA score, Sequential Organ Failure Assessment score; HB, hemoglobin; PLT, platelet; WBC, white blood cell; AG, anion gap; BC, bicarbonate; BUN, blood urea nitrogen; GLU, glucose; INR, international normalized ratio; SBP, systolic blood pressure; DBP, diastolic blood pressure; Ca, calcium; Na, natrium; K, kalium; ICU, intensive care unit; LOS, length of stay.

**Table 2.** **Univariate analysis results of the validation set**.

Variables		Non-AKI (n = 144/80.45%)	AKI (n = 35/19.55%)	*p*-value
Gender (male) (n (%))		93 (64.58)	25 (71.43)	0.571
Procedure (yes) (n (%))				
	Extracorporeal circulation auxiliary to open heart surgery	144 (100.00)	35 (100.00)	-
	Resection of vessel with replacement, thoracic vessels	109 (75.69)	31 (88.57)	0.154
	Open heart valvuloplasty of aortic valve without replacement	28 (19.44)	11 (32.43)	0.163
	Open and other replacement of aortic valve with tissue graft	27 (18.75)	5 (14.29)	0.711
	Endovascular implantation of graft in thoracic aorta	31 (21.53)	3 (8.57)	0.130
Death		7 (4.86)	2 (5.71)	1.000
Age (years)		70.02 ± 41.72	62.12 ± 13.65	0.271
Height (cm)		171.98 ± 10.36	173.01 ± 11.01	0.608
Weight (kg)		81.96 ± 18.18	93.75 ± 26.29	0.002
BMI		27.83 ± 5.43	31.00 ± 7.17	0.005
APSIII		43.52 ± 18.55	54.14 ± 16.84	0.002
GCS score		6.17 ± 15.24	5.24 ± 14.63	0.750
Charlson comorbidity index		3.94 ± 1.97	4.00 ± 2.09	0.883
SOFA score		6.05 ± 3.34	9.11 ± 3.34	0.061
HB min (g/dL)		8.28 ± 2.03	7.56 ± 1.41	0.051
HB max (g/dL)		12.54 ± 1.58	12.65 ± 1.32	0.702
PLT min (×10^9^/L)		146.40 ± 72.23	104.29 ± 42.43	0.601
PLT max (×10^9^/L)		221.54 ± 83.89	192.94 ± 61.53	0.060
WBC min (×10^9^/L)		8.15 ± 3.38	8.76 ± 3.50	0.347
WBC max (×10^9^/L)		13.08 ± 5.36	14.10 ± 5.07	0.308
AG min (mmol/L)		11.79 ± 3.13	12.94 ± 3.86	0.074
AG max (mmol/L)		14.10 ± 3.20	16.12 ± 5.02	0.014
BC min (mmol/L)		23.06 ± 2.65	21.49 ± 3.70	0.104
BC max (mmol/L)		26.01 ± 3.23	24.69 ± 3.06	0.129
BUN min (mmol/L)		17.08 ± 10.13	22.60 ± 9.10	0.004
BUN max (mmol/L)		20.58 ± 11.21	27.51 ± 10.48	0.001
Ca min (mmol/L)		0.95 ± 0.15	0.94 ± 0.13	0.610
Ca max (mmol/L)		1.28 ± 0.16	1.39 ± 0.39	0.017
Creatinine min (mg/dL)		1.12 ± 1.20	1.79 ± 2.10	0.014
Creatinine max (mg/dL)		1.37 ± 1.46	2.45 ± 2.69	0.001
GLU min (mmol/L)		94.78 ± 19.74	92.14 ± 18.28	0.474
GLU max (mmol/L)		185.51 ± 54.26	221.49 ± 64.73	0.001
Na min (mmol/L)		135.41 ± 3.14	136.14 ± 2.77	0.211
Na max (mmol/L)		141.06 ± 3.66	143.23 ± 4.13	0.103
K min (mmol/L)		3.54 ± 0.49	3.67 ± 0.55	0.168
K max (mmol/L)		5.53 ± 1.04	6.06 ± 1.13	0.108
INR min		1.19 ± 0.23	1.20 ± 0.22	0.831
INR max		1.67 ± 0.56	1.95 ± 0.78	0.068
Urine output (mL)		2004.67 ± 1323.81	904.63 ± 554.95	<0.001
SBP min (mmHg)		82.90 ± 15.05	83.80 ± 11.87	0.743
SBP max (mmHg)		146.75 ± 20.90	144.40 ± 26.50	0.573
DBP min (mmHg)		43.49 ± 7.48	44.80 ± 8.28	0.365
DBP max (mmHg)		78.65 ± 14.51	75.77 ± 11.12	0.275
Length of stay (days)		14.76 ± 10.50	17.12 ± 12.40	0.252
LOS in ICU (days)		9.39 ± 9.86	13.11 ± 12.13	0.058

AKI, acute kidney injury; BMI, body mass index; APSIII score, Acute Physiology Score III; GCS score, Glasgow coma scale score; SOFA score, Sequential Organ Failure Assessment score; HB, hemoglobin; PLT, platelet; WBC, white blood cell; AG, anion gap; BC, bicarbonate; BUN, blood urea nitrogen; GLU, glucose; INR, international normalized ratio; SBP, systolic blood pressure; DBP, diastolic blood pressure; Ca, calcium; Na, natrium; K, kalium; ICU, intensive care unit; LOS, length of stay.

### 3.2 Feature Selection in Machine Learning Models

**Fig. 2.**
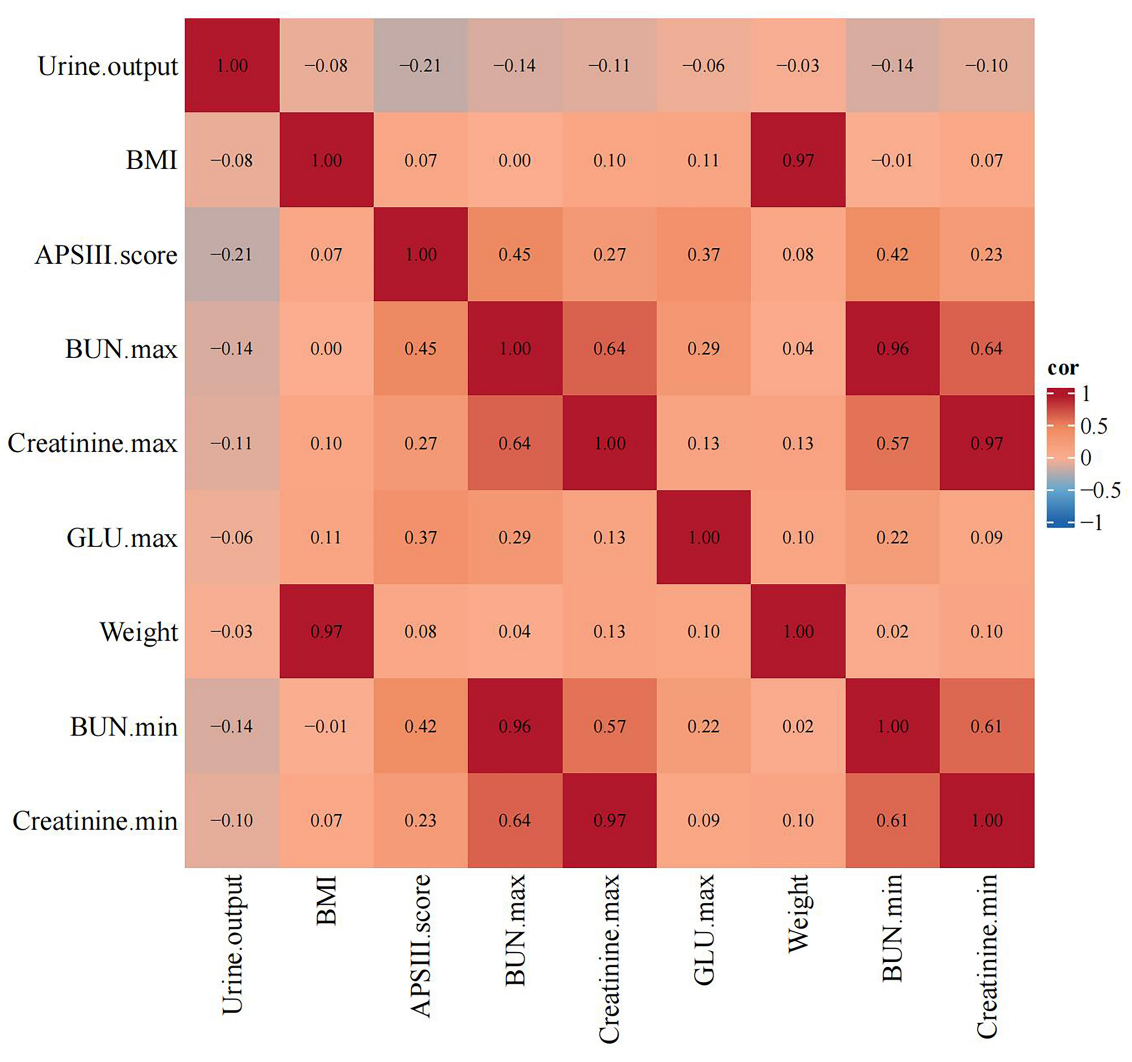
**Heat map of risk factor associations**. BMI, body mass index; APSIII, Acute Physiology Score III; BUN, blood urea nitrogen; GLU, glucose.

Feature selection for the training dataset involved univariate and multivariate logistic regression analyses. The machine learning model was built using features identified as significant risk factors with a significance level of *p* < 0.05 according to the univariate logistic regression analysis. These features included weight, BMI, APSIII score, minimum and maximum values of BUN and creatinine, maximum glucose levels, and urine output (Table 3). The correlation between all selected variables is visualized using a heat map, as shown in Fig. 2.

**Table 3.** **Results of univariate and multivariate logistic regression analyses**.

Variables		Univariable		Multivariable	
		OR (95% CI)	*p*-value	OR (95% CI)	*p*-value
Procedure (yes) (n (%))					
	Extracorporeal circulation auxiliary to open heart surgery	1.00 (1.00–1.00)	-		
	Resection of vessel with replacement, thoracic vessels	1.68 (0.66–4.30)	0.279		
	Open heart valvuloplasty of aortic valve without replacement	0.71 (0.32–1.60)	0.412		
	Open and other replacement of aortic valve with tissue graft	1.14 (0.54–2.39)	0.736		
	Endovascular implantation of graft in thoracic aorta	0.79 (0.34–1.85)	0.586		
Gender (male) (n (%))		1.52 (0.71–3.27)	0.285		
Age (years)		1.00 (0.98–1.02)	0.909		
Height (cm)		1.00 (0.98–1.03)	0.706		
Weight (kg)		1.02 (1.01–1.04)	0.002	0.98 (0.92–1.04)	0.554
BMI		1.09 (1.04–1.15)	<0.001	1.14 (0.91–1.43)	0.257
APSIII		1.03 (1.01–1.05)	<0.001	1.01 (0.99–1.03)	0.362
GCS score		1.05 (0.92–1.19)	0.502		
Charlson comorbidity index		1.08 (0.96–1.22)	0.221		
SOFA score		1.08 (0.99–1.16)	0.067		
HB min (g/dL)		0.93 (0.81–1.05)	0.248		
HB max (g/dL)		1.07 (0.92–1.23)	0.391		
PLT min (×10^9^/L)		1.00 (0.99–1.00)	0.198		
PLT max (×10^9^/L)		1.00 (1.00–1.00)	0.728		
WBC min (×10^9^/L)		1.00 (0.96–1.04)	0.948		
WBC max (×10^9^/L)		1.00 (0.98–1.02)	0.981		
AG min (mmol/L)		1.06 (0.96–1.18)	0.268		
AG max (mmol/L)		1.08 (1.00–1.17)	0.057		
BC min (mmol/L)		0.97 (0.89–1.05)	0.472		
BC max (mmol/L)		1.04 (0.95–1.13)	0.427		
BUN min (mmol/L)		1.03 (1.01–1.05)	0.011	0.90 (0.78–1.04)	0.153
BUN max (mmol/L)		1.03 (1.01–1.05)	0.001	1.10 (0.98–1.25)	0.110
Ca min (mmol/L)		0.93 (0.65–1.32)	0.647		
Ca max (mmol/L)		1.03 (0.72–1.48)	0.845		
Creatinine min (mg/dL)		1.62 (1.13–2.32)	0.008	0.43 (0.06–3.11)	0.403
Creatinine max (mg/dL)		1.98 (1.37–2.85)	<0.001	3.04 (0.57–16.04)	0.191
GLU min (mmol/L)		1.01 (1.00–1.01)	0.154		
GLU max (mmol/L)		1.01 (1.00–1.01)	0.015	1.00 (0.99–1.01)	0.918
Na min (mmol/L)		0.96 (0.90–1.04)	0.320		
Na max (mmol/L)		1.06 (0.97–1.15)	0.202		
K min (mmol/L)		1.51 (0.85–2.69)	0.159		
K max (mmol/L)		1.38 (0.97–1.96)	0.071		
INR min		0.90 (0.38–2.11)	0.706		
INR max		1.00 (0.75–1.33)	0.998		
Urine output (mL)		1.00 (1.00–1.00)	0.019	1.00 (1.00–1.00)	0.126
SBP min (mmHg)		0.99 (0.97–1.01)	0.202		
SBP max (mmHg)		0.99 (0.97–1.00)	0.135		
DBP min (mmHg)		1.00 (0.97–1.03)	0.799		
DBP max (mmHg)		1.00 (0.98–1.02)	0.926		
Length of stay (days)		1.03 (1.00–1.06)	0.068		
LOS in ICU (days)		1.03 (0.99–1.06)	0.106		

BMI, body mass index; APSIII, Acute Physiology Score III; GCS score, Glasgow coma scale score; SOFA score, Sequential Organ Failure Assessment score; HB, hemoglobin; PLT, platelet; WBC, white blood cell; AG, anion gap; BC, bicarbonate; BUN, blood urea nitrogen; GLU, glucose; INR, international normalized ratio; SBP, systolic blood pressure; DBP, diastolic blood pressure; Ca, calcium; Na, natrium; K, kalium; ICU, intensive care unit; OR, odds ratio; CI, confidence interval; LOS, length of stay.

### 3.3 Machine Learning Models in the Training Set

**Fig. 3.**
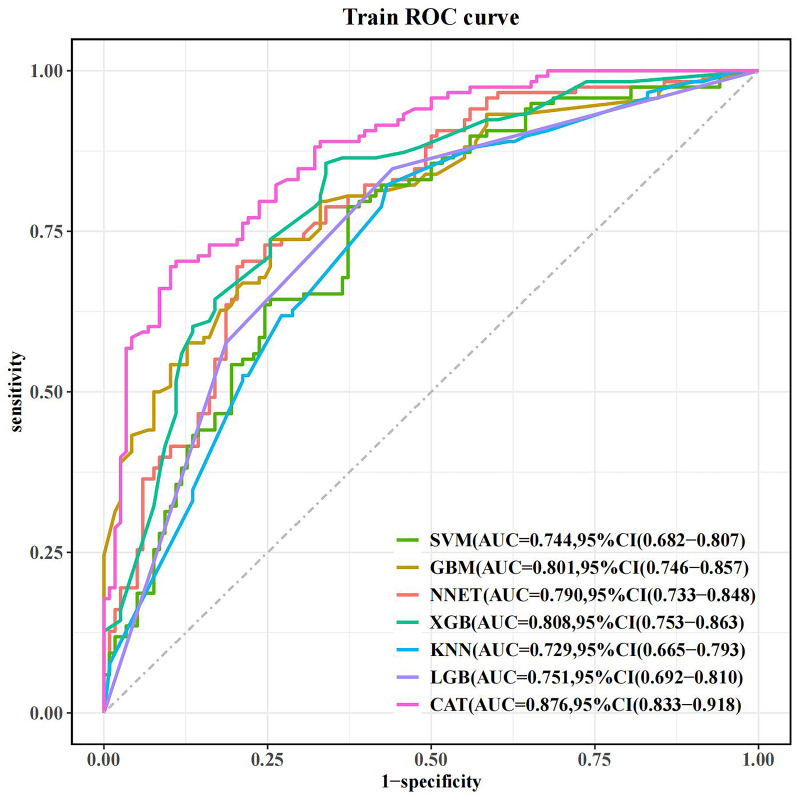
**ROC curve of the training set**. ROC, receiver operating characteristic; AUC, the area under the receiver operating characteristic curve; CI, confidence interval; SVM, support vector machine; GBM, gradient boosting machine; NNET, neural network; KNN, K-nearest neighbors; XGB, eXtreme gradient boosting; LGB, light gradient boosting machine; CAT, categorical boosting.

**Fig. 4.**
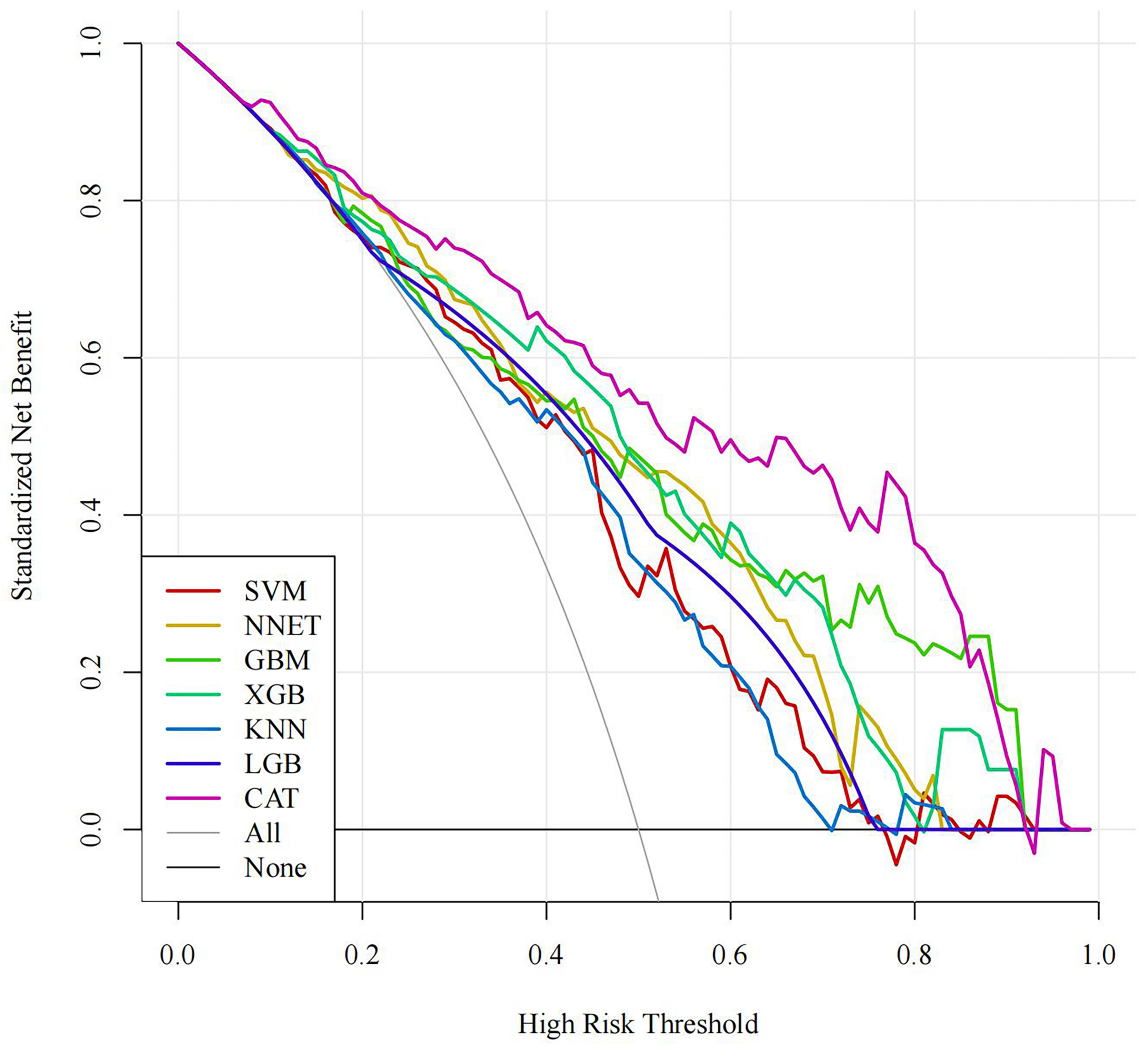
**DCA curve of the training set**. DCA, decision curve analysis; SVM, support vector machine; GBM, gradient boosting machine; NNET, neural network; XGB, eXtreme gradient boosting; KNN, K-nearest neighbors; LGB, light gradient boosting machine; CAT, categorical boosting.

**Fig. 5.**
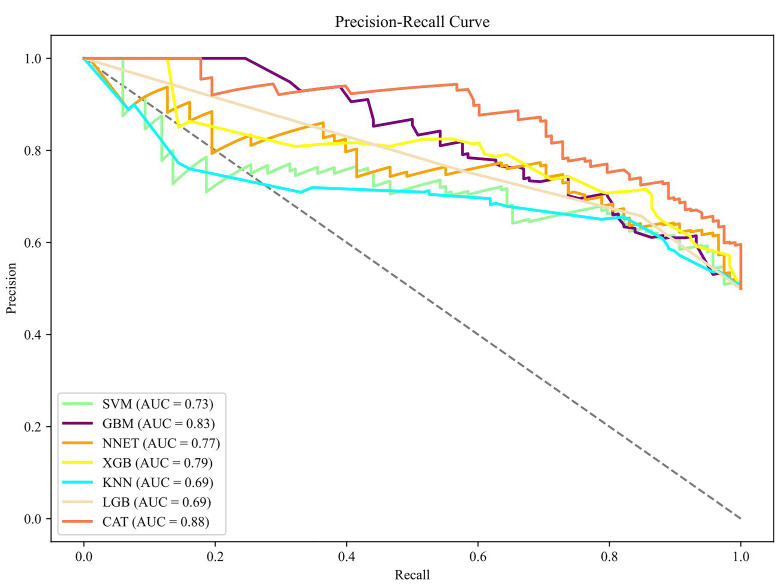
**PR curve of the training set**. SVM, support vector machine; GBM, gradient boosting machine; NNET, neural network; XGB, eXtreme gradient boosting; KNN, K-nearest neighbors; LGB, light gradient boosting machine; CAT, categorical boosting; PR, precision-recall.

**Fig. 6.**
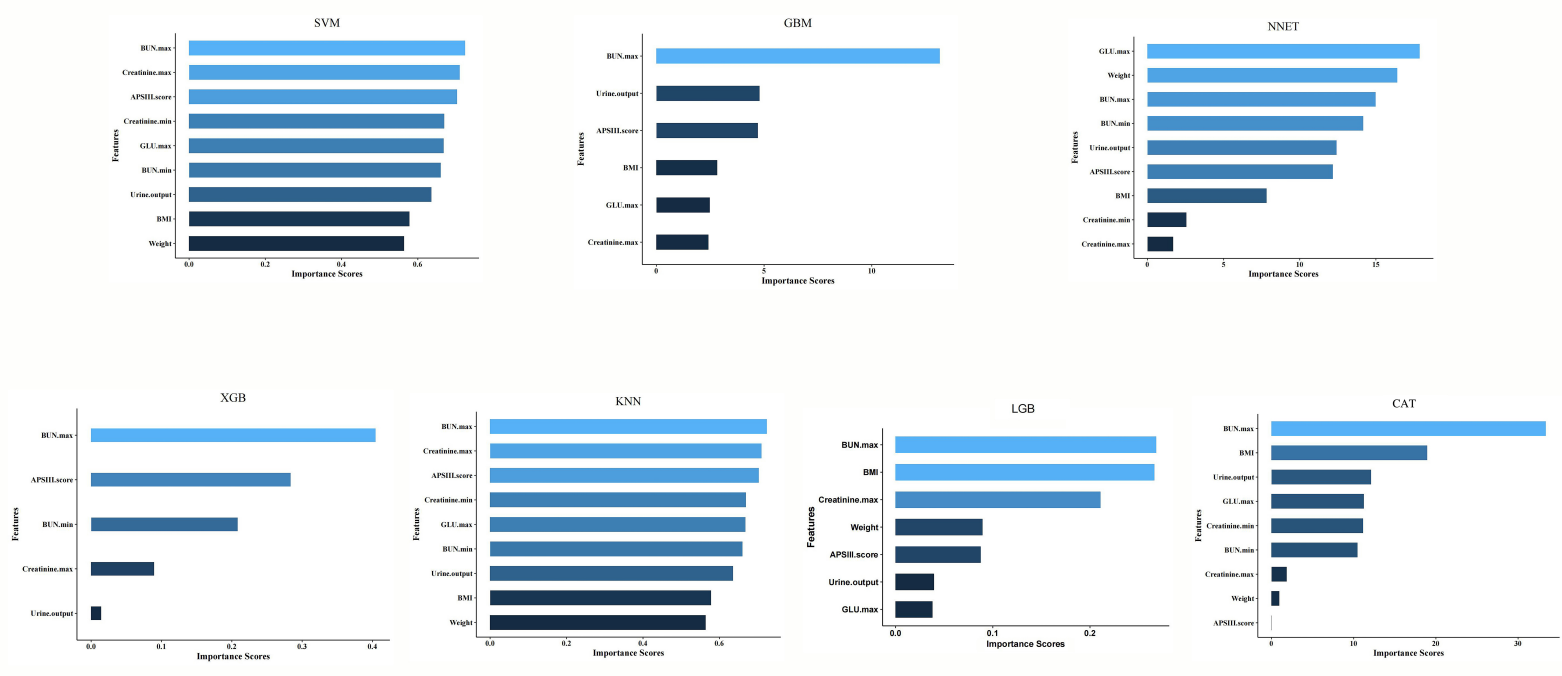
**Machine learning model characteristic importance sequence diagram**. SVM, support vector machine; GBM, gradient boosting machine; NNET, neural network; XGB, eXtreme gradient boosting; KNN, K-nearest neighbors; LGB, light gradient boosting machine; CAT, categorical boosting; BUN, blood urea nitrogen; APSIII, Acute Physiology Score III; GLU, glucose; BMI, body mass index.

Using the aforementioned features, machine learning models were constructed in the training set, and each model was ranked based on feature importance. The area under the receiver operating characteristic curve (AUC) values were as follows: CatBoost model: 0.876 (95% confidence interval (CI): 0.833, 0.918); SVM model: 0.744 (95% CI: 0.682, 0.807); GBM model: 0.801 (95% CI: 0.746, 0.857); NNET model: 0.790 (95% CI: 0.733, 0.848); XGBoost model: 0.808 (95% CI: 0.753, 0.863); KNN model: 0.729 (95% CI: 0.665, 0.793); LightGBM model: 0.751 (95% CI: 0.692, 0.810). Among all the models in the training set, the CatBoost model performed the best, while the KNN model performed the worst (Table 4). All models ROC curves are shown in Fig. 3. The DCA curve indicated that all seven machine learning models achieved clinical net benefits, as shown in Fig. 4. The PR curve demonstrated well-balanced precision and recall across all models, indicating superior performance, as observed in Fig. 5. Feature importance rankings were performed for all models, with the top 9 features for the CatBoost model being: maximum BUN, BMI, urine output, maximum GLU, minimum BUN, minimum creatinine, maximum creatinine, weight, and APSIII. The feature importance ranking for all models can be found in Fig. 6.

**Table 4.** **Machine learning model performance evaluation results**.

Model	Data	AUC	Accuracy	Sensitivity	Specificity	Precision	F1 score
SVM	Training set	0.744	0.708	0.788	0.627	0.679	0.729
	Test set	0.703	0.814	0.360	0.972	0.818	0.501
GBM	Training set	0.801	0.742	0.737	0.746	0.744	0.740
	Test set	0.703	0.691	0.600	0.722	0.429	0.500
NNET	Training set	0.790	0.746	0.703	0.788	0.769	0.735
	Test set	0.714	0.804	0.440	0.931	0.688	0.537
XGB	Training set	0.808	0.756	0.856	0.661	0.716	0.780
	Test set	0.722	0.742	0.600	0.792	0.500	0.545
KNN	Training set	0.729	0.695	0.822	0.568	0.655	0.729
	Test set	0.700	0.711	0.640	0.736	0.457	0.533
LGB	Training set	0.751	0.703	0.848	0.559	0.658	0.741
	Test set	0.639	0.670	0.560	0.708	0.400	0.467
CAT	Training set	0.876	0.797	0.695	0.898	0.872	0.774
	Test set	0.723	0.608	0.920	0.500	0.789	0.648
	Validation set	0.712	0.721	0.600	0.750	0.368	0.457

AUC, the area under the receiver operating characteristic curve; SVM, support vector machine; GBM, gradient boosting machine; NNET, neural network; XGB, eXtreme gradient boosting; KNN, K-nearest neighbors; LGB, light gradient boosting machine; CAT, categorical boosting.

### 3.4 Testing Machine Learning Models in Test Sets and Validation Sets

**Fig. 7.**
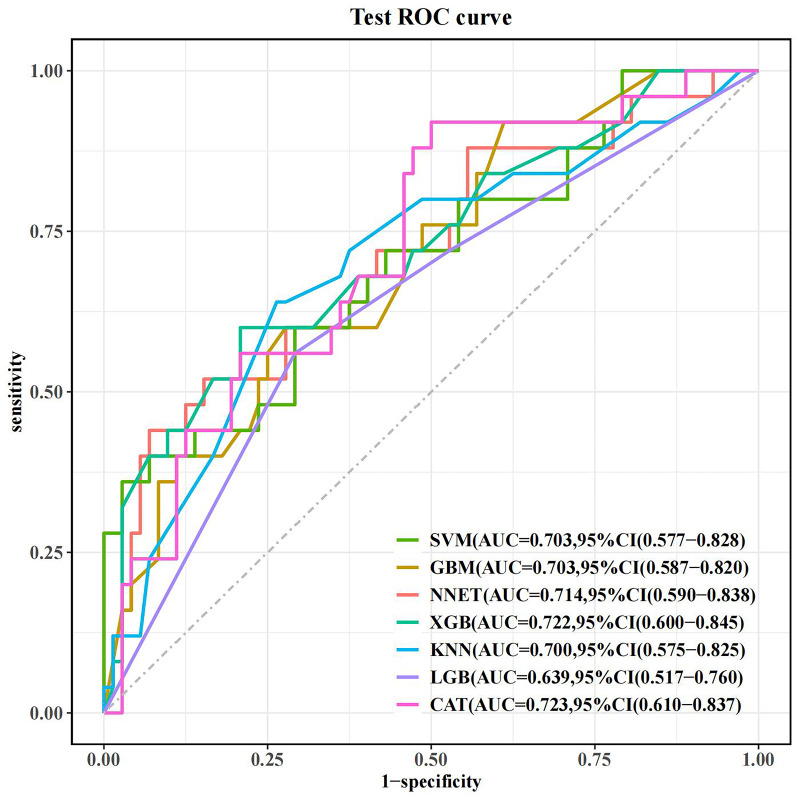
**ROC curve of the test set**. ROC, receiver operating characteristic; AUC, the area under the receiver operating characteristic curve; CI, confidence interval; SVM, support vector machine; GBM, gradient boosting machine; NNET, neural network; XGB, eXtreme gradient boosting; KNN, K-nearest neighbors; LGB, light gradient boosting machine; CAT, categorical boosting.

**Fig. 8.**
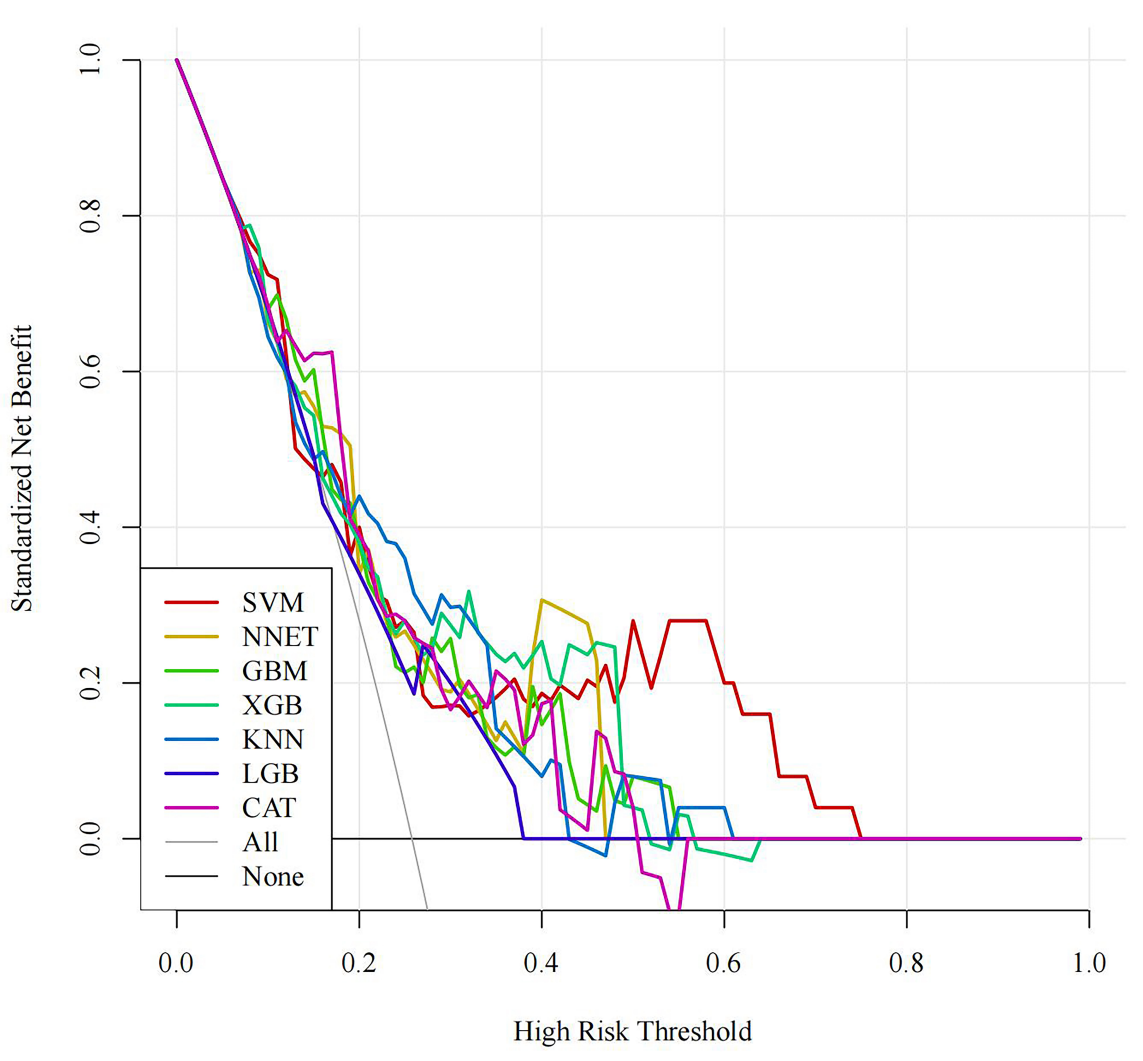
**DCA curve of the test set**. DCA, decision curve analysis; SVM, support vector machine; GBM, gradient boosting machine; NNET, neural network; XGB, eXtreme gradient boosting; KNN, K-nearest neighbors; LGB, light gradient boosting machine; CAT, categorical boosting.

**Fig. 9.**
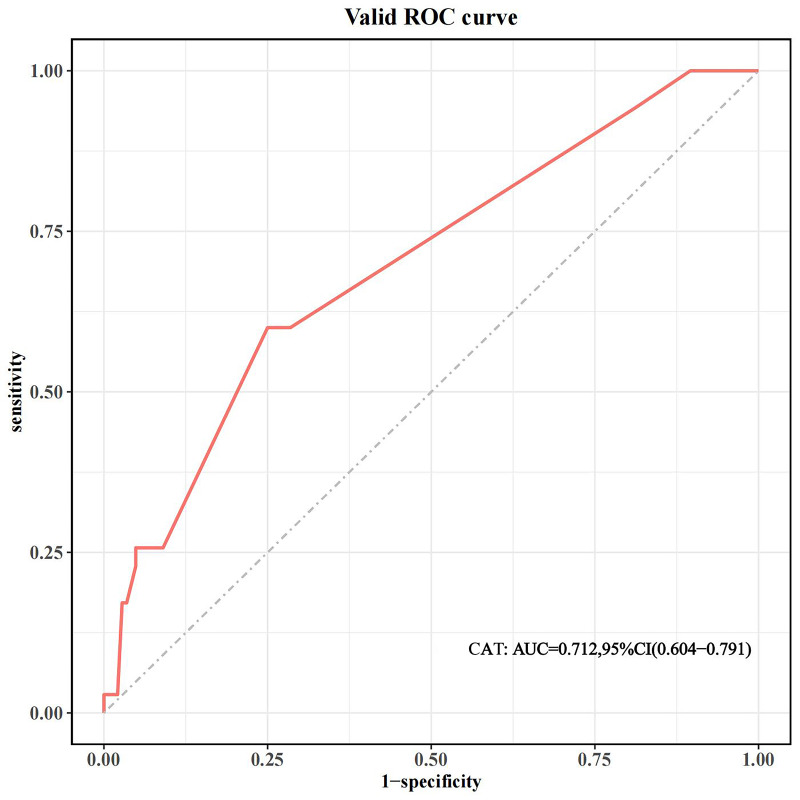
**ROC curve of the validation set**. ROC, receiver operating characteristic curve; AUC, the area under the receiver operating characteristic curve; CI, confidence interval; CatBoost, categorical boosting.

In total, 30% of the data obtained from the MIMIC-IV database were utilized as the test set to evaluate the performance of the model, assessing the AUC for each model. The AUC values obtained are as follows: CatBoost model: 0.723 (95% CI: 0.610, 0.837); SVM model: 0.703 (95% CI: 0.577, 0.828); GBM model: 0.703 (95% CI: 0.587, 0.820); NNET model: 0.714 (95% CI: 0.590, 0.838); KNN model: 0.700 (95% CI: 0.575, 0.825); LightGBM model: 0.639 (95% CI: 0.517, 0.760); XGBoost model: 0.722 (95% CI: 0.600, 0.845). Among all models in the test set, the CatBoost model demonstrated the highest AUC value, while the LightGBM model had the lowest (Table 4). The ROC curves for all models are depicted in Fig. 7, and the DCA curves in Fig. 8. The optimal model CatBoost model was externally validated using 179 patients obtained from the MIMIC-III database, which also showed good model performance with an AUC of 0.712, as shown in Fig. 9.

### 3.5 Model Interpretation

**Fig. 10.**
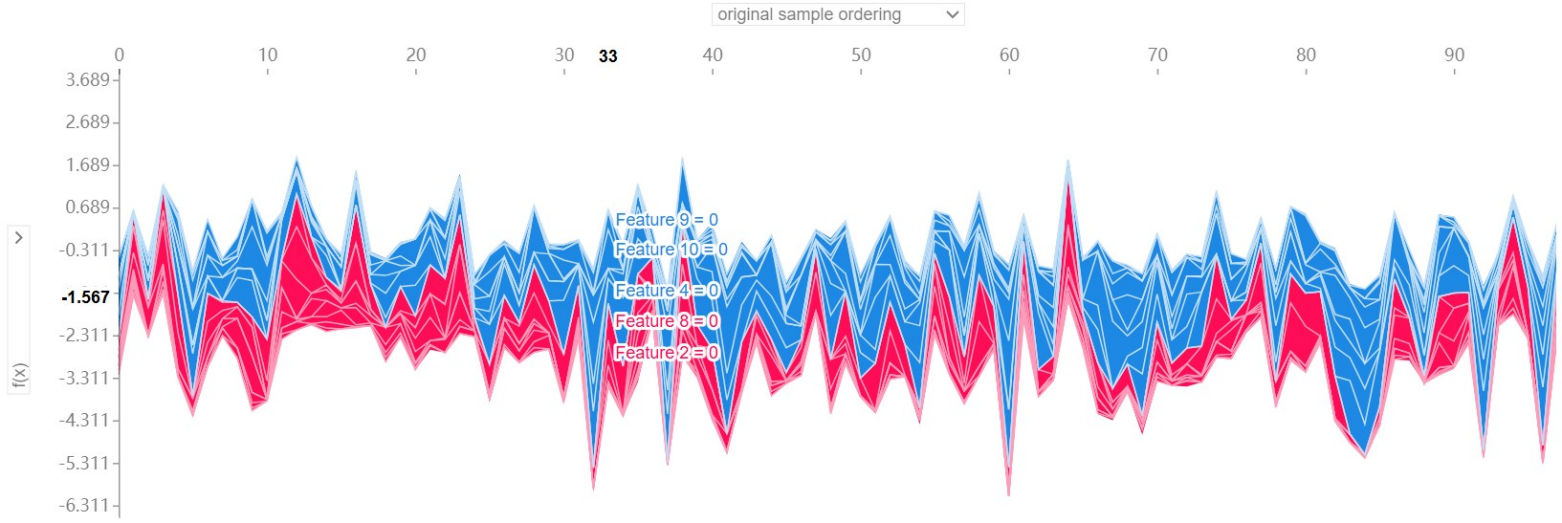
**Visualization of the overall sample characteristics**.

**Fig. 11.**
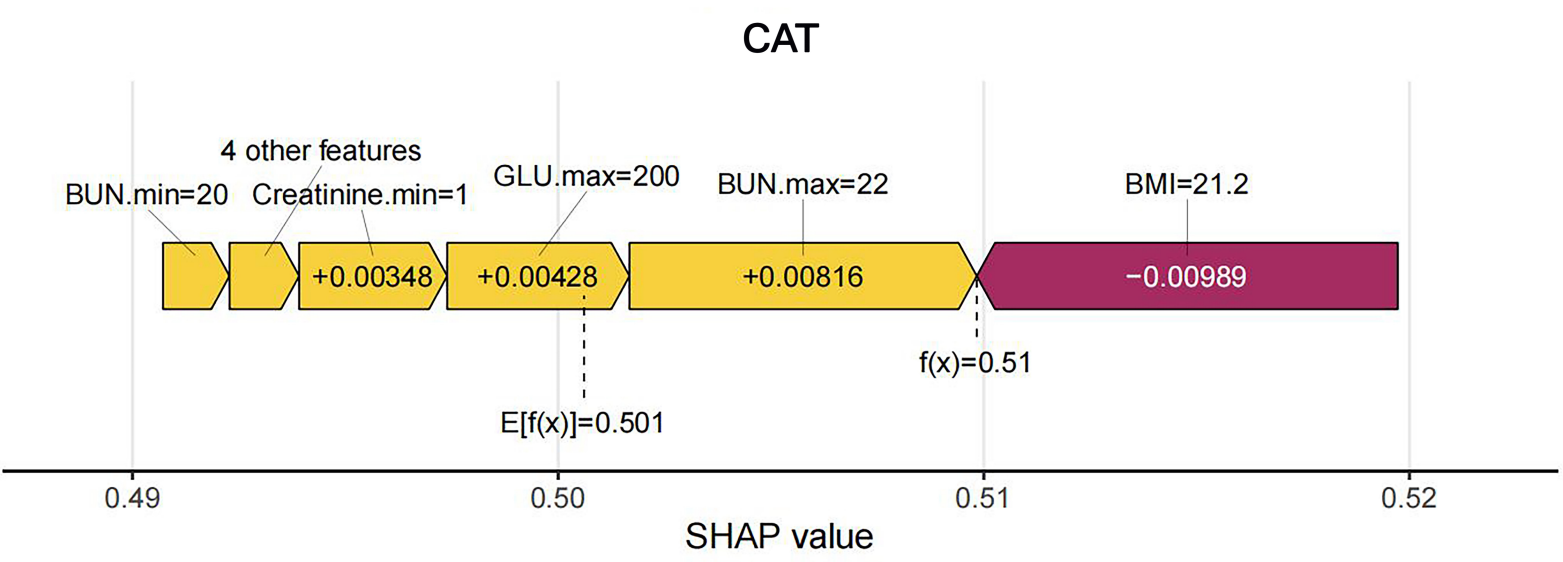
**Force plot of sample 2**. SHAP, Shapley Additive explanations; CatBoost, categorical boosting; BMI, body mass index; BUN, blood urea nitrogen; GLU, glucose.

**Fig. 12.**
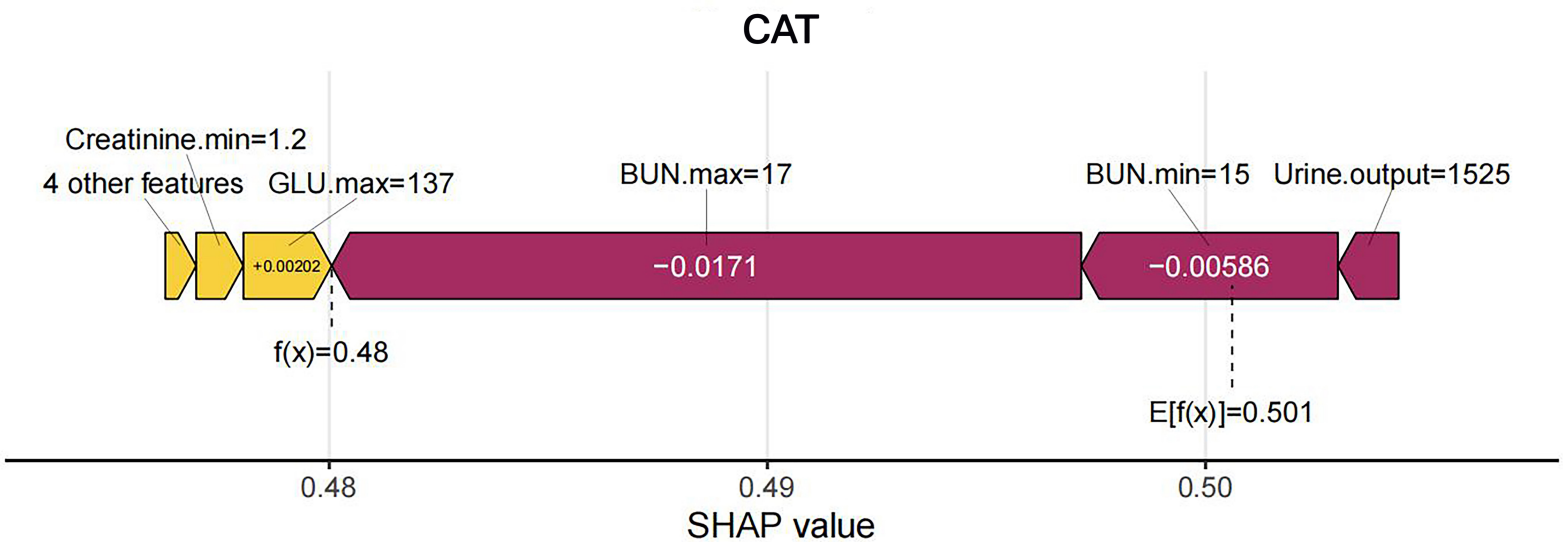
**Force plot of sample 3**. SHAP, Shapley Additive explanations; CatBoost, categorical boosting; BUN, blood urea nitrogen; GLU, glucose.

**Fig. 13.**
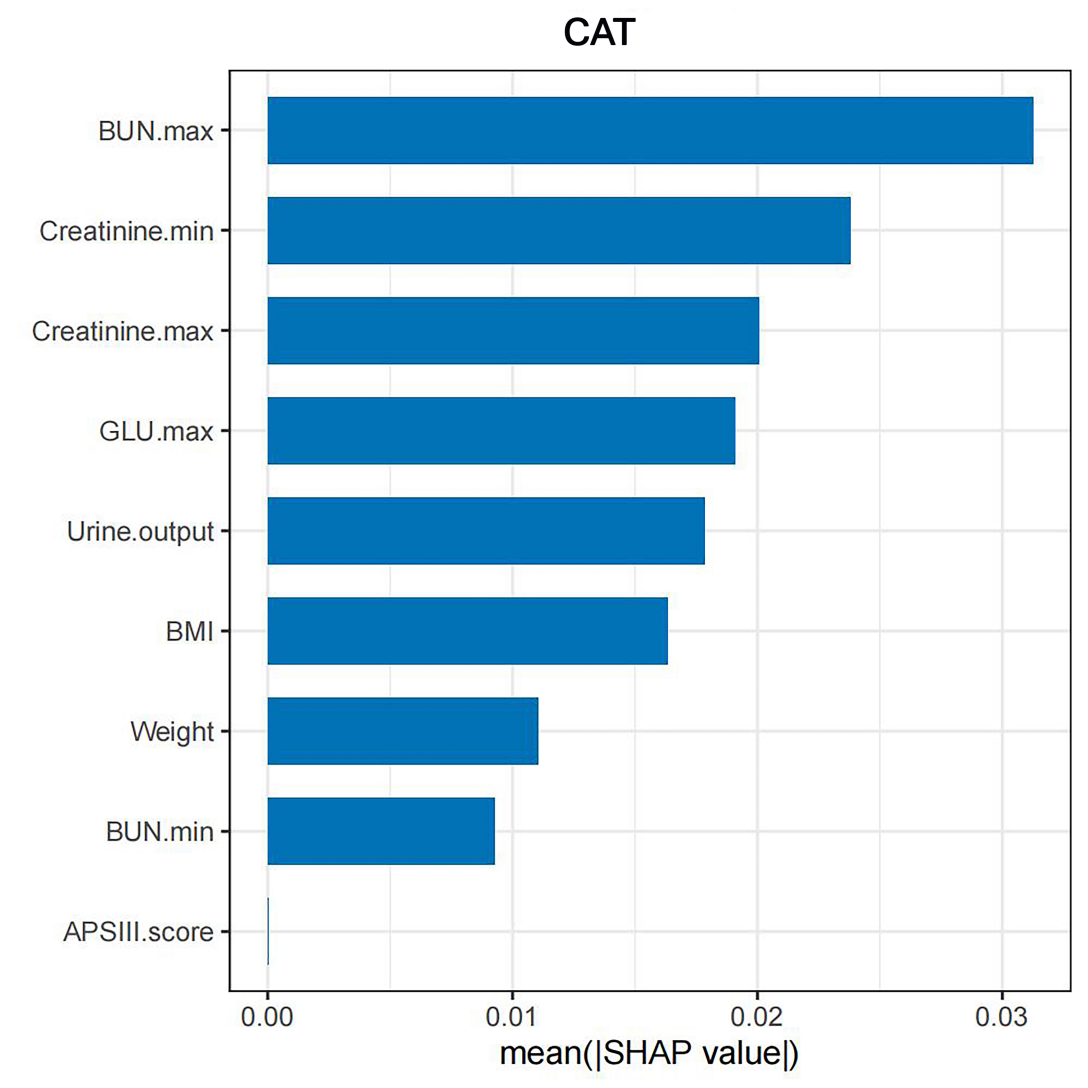
**SHAP importance ranking diagram of the CatBoost model**. SHAP, Shapley Additive explanations; CatBoost, categorical boosting; BMI, body mass index; BUN, blood urea nitrogen; GLU, glucose; APSIII, Acute Physiology Score III.

**Fig. 14.**
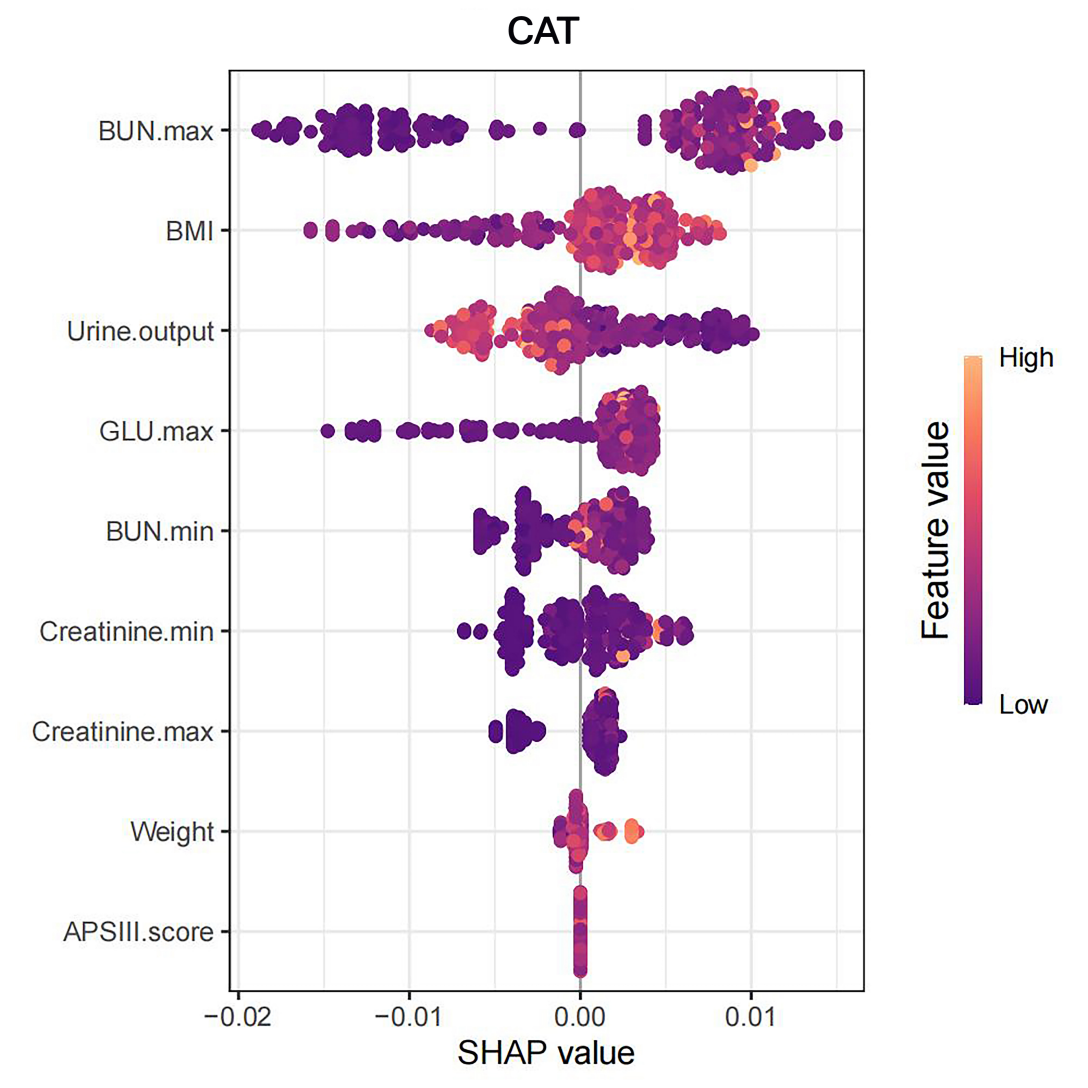
**SHAP summary plot**. SHAP, Shapley Additive explanations; CatBoost, categorical boosting; BMI, body mass index; BUN, blood urea nitrogen; GLU, glucose; APSIII score, Acute Physiology Score III.

After evaluating the model on the training, test, and validation sets, the evaluation metrics indicate that the CatBoost model performs best in this study. Therefore, the SHAP visualization method was employed to interpret the CatBoost model. Initially, the overall sample features were visualized, as shown in Fig. 10. Subsequently, force diagrams for the second and third samples were visualized. For sample 2, the final Shapley value is 0.51, with features such as maximum creatinine levels, minimum creatinine levels, maximum and minimum BUN, and urine output contributing to the increased probability of in-hospital AKI, as shown in Fig. 11. For sample 3, the final Shapley value is 0.48, with features including minimum creatinine levels, maximum creatinine levels, APSIII, and BMI contributing to the increased probability of in-hospital AKI, as shown in Fig. 12. The SHAP importance rankings and summary plots highlight the key risk factors for in-hospital AKI in AAD patients, which include maximum BUN, BMI, urine output, maximum GLU, minimum BUN, minimum creatinine, maximum creatinine, weight, and APSIII, as shown in Figs. 13,14.

## 4. Discussion

This study primarily identified the risk factors associated with AKI complications in hospitalized AAD patients. Through univariate and multivariate logistic regression analyses, clinical features including maximum BUN, BMI, urine output, maximum GLU, minimum BUN, minimum creatinine, maximum creatinine, weight, and APSIII were found to be associated with AKI occurrence during hospitalization in AAD patients. Seven machine learning models were also developed: SVM, GBM, NNET, XGBoost, KNN, LightGBM, and CatBoost. Each model exhibited unique characteristics, while performances varied across different datasets.

In this study, the CatBoost model demonstrated superior performance both in the training set (AUC = 0.876), test set (AUC = 0.723), and validation set (AUC = 0.712) compared to other models. The advantages of the CatBoost model are significant, as it can reduce prediction bias through ordered boosting and unbiased gradient estimation to combat overfitting while using diverse sampling methods to enhance both precision and accuracy, thereby enhancing the model’s generalizability. SHAP visualization analysis was employed to interpret the optimal machine learning model. The incidence of in-hospital AKI in AAD patients was 25.88%, which is consistent with previous studies [7]. Development of in-hospital AKI in AAD patients is associated with worsened outcomes and a poorer prognosis [21, 22, 23]. Therefore, the timely identification of risk factors for in-hospital AKI in AAD patients and the development of effective machine learning models are crucial for identifying high-risk patients and providing timely clinical intervention to prevent further complications.

In 2023, Dai A *et al*. [24] selected risk factors such as urine output, intraoperative hypotension, and autologous blood transfusion to establish four machine learning models, including XGBoost and SVM, to predict postoperative AKI risk in patients with AAD. However, limitations included the use of fewer models without incorporating the most recent predictive models, as well as missing external validation. In 2022, Luo CC *et al*. [25] found that variables, such as creatinine levels and extracorporeal circulation time, were closely associated with the occurrence of postoperative AKI in AAD patients; however, the researchers solely developed a nomogram, which showed lower predictive efficacy. In 2023, Zhang C *et al*. [26] selected risk factors such as hypertension and preoperative renal artery involvement and established a predictive model for in-hospital AKI in postoperative Stanford type A AAD patients, with an AUC of 0.839. However, this study had a small sample size of only 241 cases and utilized a single, simplistic model for prediction without further elaboration. Thus, the reliability of the overall research could not be guaranteed. Previous studies have not established a reliable predictive model for in-hospital AKI in Stanford type A AAD patients, thereby motivating our attempt to develop a more stable model. Utilizing SHAP visualization analysis, we interpreted the optimal CatBoost model, identifying key factors associated with in-hospital AKI, including BUN, BMI, urine output, creatinine, APSIII, etc. In AAD patients, cumulative kidney involvement often leads to renal hypoperfusion, resulting in renal impairment, decreased glomerular filtration rate (GFR), and increased renal reabsorption of water, thereby reducing urine output. Abnormal urine output in AAD patients is indicative of a higher likelihood of developing in-hospital AKI [27, 28]. BUN levels are often increased following the use of nephrotoxic drugs, potentially exacerbating kidney involvement and increasing AKI risk in AAD patients [29, 30]. Creatinine, a metabolic product of phosphocreatine and creatine in muscle tissue, is primarily filtered by the glomeruli into the urine; therefore, elevated creatinine levels often indicate impaired renal function [31, 32]. Obesity is a risk factor for various diseases, such as hypertension and hyperlipidemia [33]. Study has demonstrated that an increase of 5 kg/m^2^ in BMI increases the incidence of AKI by 40% [34], highlighting weight as an important factor in AKI occurrence. The APSIII, a severity-of-disease classification system, is commonly utilized in prognosis studies of respiratory and neurological diseases [35, 36], yet its application in AKI complications still needs to be explored. This study provides insights into the potential clinical utility of the APSIII in predicting in-hospital AKI complications in AAD patients. Based on our findings, it is recommended that clinicians actively prevent in-hospital AKI when there are notable increases in indicators such as BUN, creatinine levels, urine output, GLU, and APSIII in AAD patients or if the patient is obese. This can be performed through medication and symptomatic supportive treatment to manage elevated BUN, creatinine, and GLU levels.

## 5. Limitations

This study has several limitations: (1) the number of patients included, although retrieved under both the ICD-9 and ICD-10 coding systems for AAD diagnosis, was still insufficient, potentially leading to sampling errors and probabilistic biases; (2) our machine learning model relied on a single-center database, the MIMIC, which despite its high quality, may still contain issues such as missing data and errors; (3) the machine learning model focused exclusively on predicting in-hospital AKI in AAD patients, necessitating further research into renal complications post-discharge; (4) the incidence rate of AKI in this study data was only 25.85%, resulting in data imbalance. While oversampling techniques were employed to address these limitations, they may still compromise the effectiveness of the model.

## 6. Conclusions

We developed multiple machine learning models using data from the MIMIC-III and MIMIC-IV databases to predict in-hospital AKI in AAD patients. The CatBoost model exhibited superior performance, highlighting its potential clinical implications. This study identified several factors associated with the occurrence of in-hospital AKI in AAD patients, including maximum BUN, BMI, urine output, maximum GLU, minimum BUN, minimum creatinine, maximum creatinine, weight, and APSIII.

## Availability of Data and Materials

The data utilized in this study are accessible via the following online database link: https://physionet.org/content/mimiciv/2.2/, last accessed date on 1 June 2024.

## Supplementary Material

Supplementary material associated with this article can be found, in the online version, at https://doi.org/10.31083/RCM25768.
